# Dataset of 100-year flood susceptibility maps for the continental U.S. derived with a geomorphic method

**DOI:** 10.1016/j.dib.2017.03.044

**Published:** 2017-04-08

**Authors:** Caterina Samela, Salvatore Manfreda, Tara J. Troy

**Affiliations:** a Università degli Studi della Basilicata, Potenza 85100, Italy; b Lehigh University, Bethlehem, PA 18015, USA

**Keywords:** Flood susceptibility, Terrain analysis, Geomorphic flood index, Linear binary classifier, USA, Digital elevation models (DEMs)

## Abstract

Efficient strategies for preparing communities to protect against, respond to, recover from, and mitigate flood hazard are often hampered by the lack of information about the position and extent of flood-prone areas. Hydrologic and hydraulic analyses allow to obtain detailed flood hazard maps, but are a computationally intensive exercise requiring a significant amount of input data, which are rarely available both in developing and developed countries. As a consequence, even in data-rich environments, official flood hazard graduations are often affected by extensive gaps. In the U.S., for instance, the detailed floodplain delineation produced by the Federal Emergency Management Agency (FEMA) is incomplete, with many counties having no floodplain mapping at all. In this article we present a mapping dataset containing 100-year flood susceptibility maps for the continental U.S. with a 90 m resolution. They have been obtained performing a linear binary classification based on the Geomorphic Flood Index (GFI). To the best knowledge of the authors, there are no available flood-prone areas maps for the entire continental U.S. with resolution lower that 30׳׳×30׳׳ (approximatively 1 km at the equator).

**Specifications Table**

**Table:** 

*Subject area*	*Earth and Environmental Sciences*
*More specific subject area*	*Surface hydrology*
*Type of data*	*Raster maps*
*Data format*	*ASCII text file*
*Experimental features*	*The mapping products (100-year flood susceptibility maps) have been derived for the continental U.S. through terrain analysis and a technique of linear binary classification based on the Geomorphic Flood Index. As input data, HydroSHEDs DEMs and FEMA (Federal Emergency Management Agency) flood hazard graduation have been used.*
*Data accessibility*	*Public repository: 4TU.Centre for Research Data,*doi:10.4121/uuid:4dc7ef9f-80d0-4b78-8161-ca35f215d547, url:http://dx.doi.org/10.4121/uuid:4dc7ef9f-80d0-4b78-8161-ca35f215d547*Samela, C.*, [6].

**Value of the data**

• The maps identify areas geomorphologically prone to be inundated, and can be useful in those portions of U.S. were the FEMA flood hazard classification is lacking.
• The dataset is useful for people, companies and farmers increasing awareness and understanding of flood risk to support actions to reduce risk.
• The data may also help to determine appropriate risk-based premium rates for insurance purposes and identify interventions priorities for authorities.
• The maps may be useful for comparison purposes by researchers involved in flood hazard assessments in data scarce environments.

## 1 Data

This dataset provides binary maps of the areas exposed to flood inundation for the continental U.S. The 100-year return period has been chosen since is usually adopted in U.S. as a standard for design and in creating flood risk maps.

These mapping products were derived through terrain analysis and a technique of pattern classification performed on DEMs obtained from HydroSHEDS – the void-filled DEM, and the hydrologically conditioned DEM (http://hydrosheds.cr.usgs.gov/overview.php) – with a 3 arc-second resolution (0.00083333°, approximatively 90 m at the equator). Specifically, the flood-prone areas were identified by applying a linear binary classifier based on a morphologic descriptor named Geomorphic Flood Index (GFI) [4], [7], [8]. The raster maps have a 90 m resolution and are geo-referenced. The coordinate system of the maps is UTM (Universal Transverse Mercator) Zone 17N, the projection is Transverse Mercator, and the geodetic system is NAD (North American Datum) 1983.

**Fig. 1:**
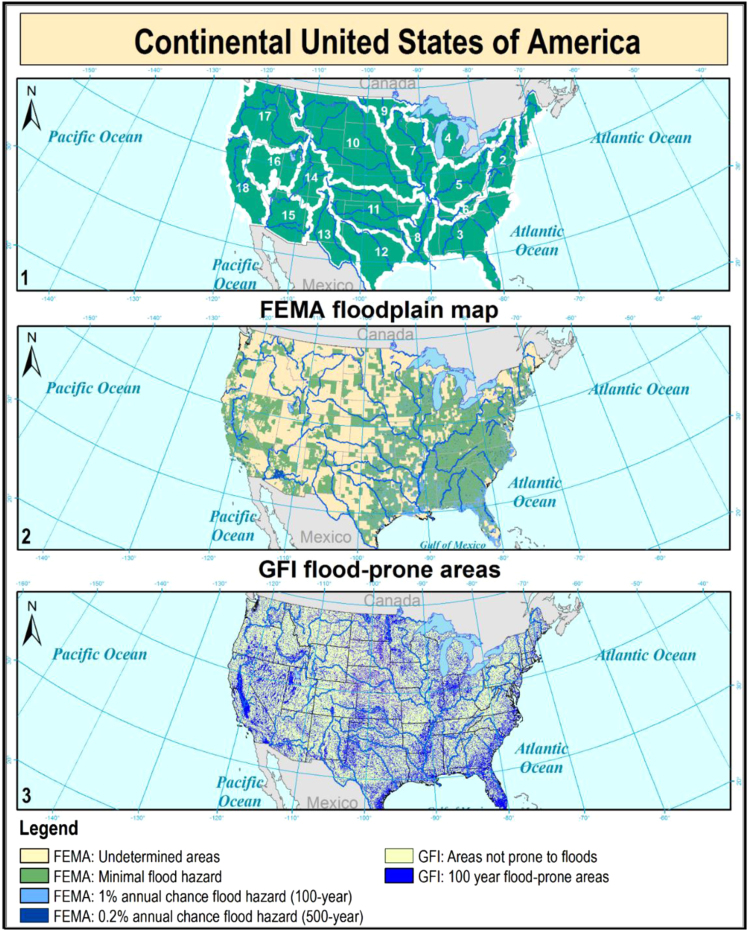
Panel 1 displays the 18 water resources regions in which the continental U.S. and the present dataset have been divided; panel 2 synthesizes FEMA flood hazard classification, showing areas where the hazard is still undetermined; panel 3 depicts the 100-year flood susceptibility maps obtained using the GFI linear binary classifier.

To simplify the management and the use of the data, the continental U.S. was divided into 18 major water resources regions, considering the hydrologic units identified by the United States Geological Survey (USGS) (see Fig. 1, panel 1).

## 2 Experimental design, materials and methods

The flood-prone areas maps have been derived applying a technique of pattern recognition through computational learning. Starting from morphological attributes of a basin, embodied in the Geomorphic Flood Index, a linear binary classifier was built and used to delineate flood-prone areas according to the procedure proposed by Manfreda et al. [2], [3], [4] and Samela et al. [7], [8].

To extract the fluvial geomorphology, the DEMs of HydroSHEDS (Hydrological Data and Maps Based on Shuttle Elevation Derivatives at Multiple Scales) developed by the Conservation Science Program of the World Wildlife Fund have been used. Specifically, the DEM-Void and DEM-CON with a spatial resolution of 3 arc-second have been used; the area was then regridded with a cell size of 90 m.

Flow direction and flow accumulation patterns were derived from the 90-m resolution DEM-CON to obtain a spatially uniform stream network for the continental U.S. Then, the procedure proposed by Giannoni et al. [1] for the identification of the drainage network from geomorphology has been carried out. It relies on a combination of contributing area and slope as a criterion for drainage network extraction.

Considering the significant extent of the study area, the analyses were performed sub-dividing the Country according to the 18 water-resources regions identified in the continental U.S. by USGS (http://water.usgs.gov/GIS/huc_name.html). These geographic areas contain either the drainage area of a major river or the combined drainage areas of a series of rivers, and are depicted in Fig. 1, panel 1.

For each major region, the GFI binary classifier have been trained using the 1-percent-annual-chance flood event (return period of T=100 year) of the Federal Emergency Management Agency׳s (FEMA) Flood Insurance Rate Maps (FIRMs) [5]. The mentioned dataset has limitations, as shown in Fig. 1, panel 2, since FEMA maps contain several gaps in different zones of the U.S. where there are possible but undetermined flood hazards, since no analysis (neither with detailed or approximate methods) has been conducted.

To generate these susceptibility flood maps, the full available floodplains determined with detailed methods of analysis have been used to train the geomorphic classifiers (i.e. FEMA׳s FIRMs Zones AE, A1-A30, AH, AO). The portions of the flood map having coastal floodplains with additional hazards associated with storm waves (FEMA zones V, VE) have not been considered for calibration and validation, since this kind of morphologic analysis cannot take into account storm surge that may result in flooding of coastal areas. Any “Undetermined area”, “Open Water”, “Area not Included’ have been removed and not considered as calibration area.

To carry out the binary classification, the standard flood hazard maps have been converted into binary maps, where the value 0 represents the areas not prone to floods (Area of Minimal Flood hazard) and the value 1 represents the 100 year floodplains. After being calibrated in a region, each GFI classifier has been applied within that water resources region. The obtained geomorphologic flood-prone areas for the continental U.S. are shown in Fig. 1, Panel 3.

### 2.1 Dataset specification and metadata

Raster data format: ASCII text file *.txt.

Raster resolution: 90 m.

Coordinate system: Transverse Mercator projection, NAD (North American Datum) 1983 geodetic system, UTM (Universal Transverse Mercator) coordinate system - Zone 17N.
